# Long-Term Road Traffic Noise, Air Pollution, and Cardiovascular Disease AIRCARD: A Prospective Cohort Study

**DOI:** 10.1016/j.jacadv.2025.101787

**Published:** 2025-05-21

**Authors:** Stephan P. Mayntz, Roda A. Mohamed, Anna Mejldal, Jens-Jakob K. Møller, Jes S. Lindholt, Axel CP. Diederichsen, Lise M. Frohn, Jørgen Brandt, Matthias Ketzel, Jibran Khan, Jess Lambrechtsen

**Affiliations:** a Cardiology Research Unit, Odense University Hospital, Svendborg, Denmark; b Department of Clinical Research, University of Southern Denmark, Odense, Denmark; c OPEN – Open Patient Data Explorative Network, Odense University Hospital, Odense, Denmark; d Department of Cardiothoracic and Vascular Surgery, Odense University Hospital, Odense, Denmark; e Department of Cardiology, Odense University Hospital, Odense, Denmark; f Department of Environmental Science, Aarhus University, Roskilde, Denmark

**Keywords:** air pollution, cardiovascular disease risk, environmental pollution, prospective cohort study, road traffic noise

## Abstract

**Background:**

Air pollution and road traffic noise are major environmental stressors associated with cardiovascular disease (CVD). Although their independent effects are well-documented, few studies have concurrently evaluated their relative contributions—particularly in low ambient air pollution settings.

**Objectives:**

The purpose of this study was to investigate the association between long-term exposure to air pollution and road traffic noise and CVD.

**Methods:**

We conducted a prospective cohort study using data from the DANCAVAS (Danish Cardiovascular Screening Trial) (2014-2018) and VIVA (Viborg vascular) (2008-2010) screening trials, including 26,723 men aged 65 to 74 years with pollution exposure data from 1979 to 2019. Residential exposure to air pollutants (particulate matter with a diameter <2.5 μm, nitrogen dioxide, warm-season ozone, sulfur dioxide, and carbon monoxide) and road traffic noise (L_den_) were estimated based on residential addresses using the Danish Eulerian Hemispheric Model/Urban Background Model/Air Geographic Information System model and Nord2000. We used Cox proportional hazards models, adjusting for baseline inclusion year, individual-level lifestyle factors, family history of CVD, and socioeconomic status. Major adverse cardiovascular events was the primary outcome.

**Results:**

A 14.9 decibel increase in road traffic noise (IQR increment) was associated with a 7.5% higher risk of major adverse cardiovascular events (HR: 1.075; 95% CI: 1.026-1.128) and an 8.1% increased risk of all-cause mortality (HR: 1.081; 95% CI: 1.027-1.137). No significant associations were found between air pollutants and the primary or secondary outcomes in the pooled cohort after adjustment for confounders.

**Conclusions:**

Long-term exposure to road traffic noise was significantly associated with increased CVD and all-cause mortality. Our findings suggest that studies that do not consider noise exposure may overestimate the cardiovascular burden attributed to air pollution. (Impact of Lifetime Exposure to Air and Noise Pollution on Cardiovascular Disease and Mortality-the AIRCARD Study [AIRCARD]; NCT04353232)

## Highlights

• Environmental noise and air pollution are critical contributors to cardiovascular disease risk in aging populations.
• Road traffic noise independently increases cardiovascular risk, highlighting the importance of noise-reduction interventions.
• Although air pollution remains a global health concern, noise exposure independently contributes to cardiovascular risk.
• Future studies should evaluate targeted noise mitigation strategies to effectively reduce cardiovascular disease burden.

Air pollution is a critical public health challenge in both low- and middle-income countries and high-income countries (HICs) where cardiovascular disease (CVD) is a leading cause of morbidity and mortality.^1^^,^^2^ In 2021, air pollution became the world’s second-largest mortality risk factor, surpassing tobacco and highlighting its significant role in CVD.^3^ Even as global levels of fine particulate matter with a diameter <2.5 μm (PM_2.5_) decrease or stabilize, populations in many HICs remain exposed to PM_2.5_, nitrogen dioxide (NO_2_), and ozone (O_3_) at levels posing substantial cardiovascular (CV) risks.^3^ CVD inflicts a substantial economic burden, straining health care systems, and reducing productivity.^4^

PM_2.5_ and NO_2_ primarily originate from vehicular emissions, industrial activities, and residential heating, while O_3_ forms secondarily through photochemical reactions involving nitrogen oxides and volatile organic compounds.^5^ These pollutants contribute to CVD via mechanisms such as oxidative stress, systemic inflammation, endothelial dysfunction, and accelerated atherosclerosis.^6^^,^^7^ Epidemiological studies have linked long-term air pollution exposure to increased risks of myocardial infarction, stroke, heart failure, and CVD mortality.^8^, ^9^, ^10^ Despite pollution reduction efforts, recent studies show that even low-level exposures can adversely affect health.^3^ This persistent exposure necessitates a better understanding of air pollution’s impact on CV health, especially among aging populations who are particularly vulnerable.^11^ Environmental noise, particularly from road traffic, appears to be an independent risk factor for CVD.^12^, ^13^, ^14^ Chronic exposure to noise pollution has been linked to increased risks of hypertension, myocardial infarction, and heart failure.^15^^,^^16^ Road traffic noise is a pervasive environmental stressor in urban environments, originating from vehicular engines, tire-road interactions, and aerodynamic friction.^17^, ^18^, ^19^ Noise pollution contributes to CVD through biological mechanisms involving stress responses, hormonal imbalances, sleep disturbances, and autonomic dysfunction.^20^ Epidemiological evidence suggests that noise exposure is associated with adverse CV outcomes, independent of air pollution exposure.^21^ In urbanized areas, air pollution and road traffic noise often share common sources, primarily vehicular traffic, and are frequently colocalized. This co-occurrence could lead to additive or even synergistic effects on CV health.

Even with the well-documented health impacts, most research investigates either air pollution or noise pollution individually, rather than assessing both exposures within the same framework.^22^ This approach limits understanding of how each pollutant independently contributes to CVD risk.^23^ However, existing studies are limited by a lack of confounding factors, exposure assessment inaccuracies, and absence of long-term data, emphasizing the need for comprehensive research that evaluates both exposures concurrently.^10^^,^^24^

The AIRCARD (AIR pollution and CARDiovascular disease) study aimed to address these gaps by investigating the long-term effects of cumulative air pollution and road traffic noise exposure on CV health within the context of a high-income country.^25^ Drawing on data from the DANCAVAS (Danish Cardiovascular Screening Trial) and VIVA (Viborg vascular) screening trials,^26^ this prospective cohort study combined air and noise pollution exposure assessments with CVD. By utilizing high-resolution exposure modeling and detailed individual-level health data and national registries, this study evaluated the independent effects of air pollution and road traffic noise on CVD.

## Methods

### Study population

This prospective cohort study included 26,723 men aged 65 to 74 years, selected from the Danish DANCAVAS and VIVA screening trials. The VIVA trial recruited participants between 2008 and 2010, and the DANCAVAS trial recruited participants between 2014 and 2018. Both trials were population-based and randomized and have been described previously.^27^^,^^28^ Data harmonization was straightforward as both cohorts were highly comparable in design, recruitment area, and data collection methods. We combined the cohorts by merging individual-level data from national registries and modeling exposures for each participant. Any overlap—where individuals were enrolled in both trials—was resolved by assigning them to the VIVA cohort. Participants who had CVD at baseline were excluded from the study. We combined individual-level data from these cohorts with national registries via Statistics Denmark to cover sociodemographic information, prescription history, medical records, and mortality. We obtained the residential addresses between 1979 and 2019 of each participant from the Danish Civil Registration System and geocoded 99.98% of these.^29^

The DANCAVAS and VIVA studies were approved by the National Research Ethics Committee (S20140028, S20160164, and M20080028). Access to the participants’ former residential addresses has been approved by the Danish Health Data Authority (FSEID-00005213).

We adhered to the Strengthening the Reporting of Observational Studies in Epidemiology reporting guideline.

## Exposure assessment

### Air pollution

To assess the concentrations of PM_2.5_, NO_2_, warm-season O_3_, sulfur dioxide (SO_2_), and carbon monoxide (CO) at each participants’ address from 1979 to 2019, we utilized the Danish state-of-the-art integrated air pollution modeling system Danish Eulerian Hemispheric Model (DEHM)/Urban Background Model (UBM)/Air Geographic Information System.^30^^,^^31^ This advanced modeling system calculates air pollution concentrations at each address point in Denmark by incorporating contributions to air pollution levels from the regional background, local background, and street-level emissions, yielding ambient air pollution concentrations at high temporal and spatial resolutions. The regional background was modeled using the DEHM, which covers the Northern Hemisphere with 4 nested domains, with the innermost domain having a resolution of 5.6 km × 5.6 km over Denmark.^32^, ^33^, ^34^ The local background was modeled using the UBM at a resolution of 1 km × 1 km over Denmark, capturing emissions from all local sources, such as traffic, residential heating, local industry, and power plants.^31^^,^^35^^,^^36^ Street-level pollution was calculated using the Operational Street Pollution Model, which estimates air pollution from traffic on individual streets based on traffic data, street geometry, and meteorological conditions.^37^ An emission inventory for Denmark, including major emission categories classified by Selected Nomenclature for Air Pollution codes, was applied to model air pollution concentrations from local road traffic, residential wood combustion, agriculture, power production, shipping, and other sources.^38^ The modeling system provided hourly concentrations of all studied air pollutants at each address, from which we calculated the monthly average concentrations for each pollutant and each participant.

The DEHM/UBM/Air Geographic Information System system has been validated against measured values, demonstrating good to excellent performance. The correlation between annual mean modeled concentrations and measurements was up to 0.89 for NO_2_ and up to 0.94 for PM_2.5_.^30^

### Road traffic noise

Road traffic noise was calculated at the most exposed façade and at dwelling height, using the Nordic Prediction Method and Nord2000 in the SoundPLAN Nordic software version 8.2.^39^^,^^40^ Noise was calculated for the same geocoded residential addresses as for air pollution.

The input variables for road traffic noise included receptor points, geocoded coordinates for each residential location, and 3-dimensional building polygons for all Danish buildings. Building polygons were obtained from the Danish Geodata Agency. The Danish Road Network, which contains information on the Annual Average Daily Traffic, vehicle type distribution, traffic speed, and road type, was used in the noise calculation.

Road traffic noise was calculated for the most exposed façade of the dwelling as weekday and weekend averages, L_den_ (day, evening, night), given in decibel (dB)A. Road network attributes were obtained from the National Road Traffic database.^41^ L_den_ is a European Commission-recommended health-relevant noise metric, which includes a 5 dB and 10 dB penalties for evening and night noise levels. Noise calculations were performed from 1979 to 2019 at 5-year intervals. For the years in between, linear interpolation was performed.

## Outcomes

Our primary outcome was a composite of major adverse cardiovascular events (MACE) including nonfatal acute myocardial infarction (AMI) (10th revision of the International Classification of Diseases codes I21), nonfatal stroke (I61-65), CV mortality (I00-I99), cardiac revascularization (Nordic Medico-Statistical Committee codes KFN [A-E+G]), and peripheral revascularization (codes are provided in the Supplemental Appendix).

Secondary outcomes included individual analyses of AMI, stroke, CV mortality, cardiac and peripheral revascularization procedures, heart failure, and all-cause mortality.

### Covariates

We included the following covariates: age, smoking status, body mass index (BMI), hypertension, type 2 diabetes mellitus (T2DM), lipid-lowering medication use, family history of CVD, marital status (measured at 60 years of age), household wealth index (annual income at age 60 years, adjusted to the 2015 level to account for inflation and increasing wealth), and educational level. Age, smoking status, BMI, family history of CVD, and socioeconomic status (measured by marital status and the household wealth index) were treated as confounders, given their established influence on both pollution exposure and CV outcomes. Hypertension, T2DM, and hypercholesterolemia were considered mediators, which reflect their roles in the causal pathway from pollution exposure to CVD. Educational level and occupational status were not adjusted for because of the uniformity and retirement status of the study population. A Directed Acyclic Graph was used to guide the selection of confounders (Supplemental Figure 1).

### Statistical analysis

We applied Cox proportional hazard models to evaluate the relationship between long-term exposure to air pollution, road traffic noise, and CV outcomes. We used age as the underlying time scale to analyze time-to-event outcomes. PM_2.5_, NO_2_, warm-season O_3_, CO, SO_2_, and road traffic noise were modeled independently as time-dependent variables. Multipollutant models were not included due to the risk of collinearity. For noise, we calculated the time-weighted averages of exposure to road traffic noise at the most exposed façades at 5-year intervals between 1979 and 2019. For air, we calculated a 29-year sliding average for every month following inclusion, until death or censoring. For any given month, this measure reflects the mean exposure over the preceding 29 years. Follow-up was until the first occurrence of a CV event, death, or end of the study period. The relationship between pollutant levels and the endpoints was quantified using HR with 95% CI. All HRs are reported per IQR increase in pollutant levels. The proportional hazards assumption was verified using Schoenfeld residuals.

We fitted a series of models for the primary outcome with progressively comprehensive levels of adjustment for confounders, selected based on theoretical considerations, and a priori construction of a Directed Acyclic Graph to address confounding (Supplemental Appendix). Three models were developed. Model 1 included adjustments for baseline inclusion year to account for temporal trends and age-related effects. Model 2 was further adjusted for individual-level confounders (BMI, smoking status, and family history of CVD). Model 3 was the fully adjusted model, incorporating additional socioeconomic status.

For the secondary outcomes, similar Cox proportional hazards models were employed. These analyses followed the same modeling strategy as the primary outcome, adjusting for the same confounders across the 3 models.

To account for competing risks, particularly the risk of non-CV mortality, we supplemented with a Fine and Gray competing risk model. Spearman’s rank correlation analysis was conducted to evaluate the strength and direction of the associations between pairs of pollutants. Additionally, a dose-response relationship was created to explore the gradient of risk associated with increasing levels of road traffic noise.

We anticipated minimal missing data given the comprehensiveness of the National Registries and the limited gaps in our modeled pollution data. Similarly, the VIVA and DANCAVAS data sets showed limited missing data for variables of interest. Therefore, we decided to tolerate missing data up to a 5% threshold without intervention.

Statistical analyses were conducted using Stata (STATA/MP18.0, StataCorp LLC) on the Statistics Denmark’s Research Service Data Portal.

The Statistical Analysis Plan for this study has been published in detail elsewhere.^26^ Minor post hoc publication changes were made and are discussed in Supplemental Appendix.

### Patient and public involvement

Patients and their families were involved from the early stages of the DANCAVAS and VIVA trials. Their priorities, experiences, and preferences informed the development of the research questions and outcome measures. For the present study, patients contributed to the study design by identifying outcomes of particular interest and advising on effective methods for disseminating the findings to participants and the wider community.

## Results

The pooled study cohort included 26,723 male participants aged 65 to 74 years from the Danish DANCAVAS and VIVA screening trials (Table 1). The mean age of the participants was 69.0 years. The cohort had a high prevalence of CV risk factors; 79.2% had hypertension, 10.5% had T2DM, and 31.5% were on lipid-lowering medications. Smoking history varied, with 30.8% never having smoked, 50.1% being former smokers, and 19.1% being current smokers. BMI was also notable, with 69.6% of the participants being overweight or obese (BMI ≥25 kg/m^2^).

**Table 1:** Individual-Level Characteristics Including Demographics, CV Risk Factors, Socioeconomic Status, Medication Use, and Exposure

	VIVA Cohort (n = 18,199)	DANCAVAS Cohort (n = 8,524)	Pooled Cohort (n = 26,723)	*P* Value
Age, mean (SD), y	69.0 (2.8)	68.9 (2.6)	69.0 (2.7)	0.008
Male, %	100	100	100	
MACE, n	4.115 (22.6%)	396 (4.6%)	4.511 (16.9%)	<0.001
Mean follow-up, y (SD)	8.3 (3.0)	3.3 (0.9)	6.7 (3.5)	<0.001
Smoking status, n (%)				<0.001
Never	5,362 (29.5%)	2,839 (33.4%)	8,201 (30.8%)	
Former	8,967 (49.4%)	4,404 (51.8%)	13,371 (50.1%)	
Current	3,838 (21.1%)	1,255 (14.8%)	5,093 (19.1%)	
BMI, mean (SD)	26.8 (3.8)	27.8 (4.2)	27.2 (4.0)	<0.001
BMI, n (%)				
Underweight (<18.5)	71 (0.4%)	23 (0.3%)	94 (0.4%)	
Normal (≥18.5-<25)	5,870 (32.7%)	2,068 (24.3%)	7,938 (30.0%)	
Overweight (≥25-<30)	8,861 (49.4%)	4,254 (49.9%)	13,115 (49.6%)	
Obese (>30)	3,125 (17.4%)	2,175 (25.5%)	5,300 (20.0%)	
Comorbidities				
Hypertension, n (%)				<0.001
No	3,013 (16.6%)	2,512 (29.7%)	5,525 (20.8%)	
Yes	15,103 (83.4%)	5,956 (70.3%)	21,059 (79.2%)	
T2DM, n (%)				0.089
No	16,220 (89.3%)	7,670 (90.0%)	23,890 (89.5%)	
Yes	1,944 (10.7%)	854 (10.0%)	2,798 (10.5%)	
Family history of CVD, n (%)				<0.001
No	14,853 (81.6%)	4,814 (56.5%)	19,667 (73.6%)	
Yes	3,346 (18.4%)	3,710 (43.5%)	7,056 (26.4%)	
Medication, n (%)				
Lipid-lowering agents				<0.001
No	11,572 (66.1%)	6,269 (73.5%)	17,841 (68.5%)	
Yes	5,944 (33.9%)	2,255 (26.5%)	8,199 (31.5%)	
Marital status, n (%)				<0.001
Single	864 (4.8%)	451 (5.3%)	1,315 (4.9%)	
Married	15,284 (84.0%)	7,101 (83.3%)	22,385 (83.8%)	
Divorced	1,481 (8.1%)	764 (9.0%)	2,245 (8.4%)	
Widowed	535 (2.9%)	208 (2.4%)	741 (2.8%)	
Unknown	35 (0.2%)	0 (0.0%)	37 (0.1%)	
Household wealth index, n (%)				0.053
Quintile 1	3,688 (20.3%)	1,637 (19.3%)	5,325 (20%)	
Quintile 2	3,647 (20.1%)	1,678 (19.8%)	5,325 (20%)	
Quintile 3	3,564 (19.7%)	1,760 (20.7%)	5,324 (20%)	
Quintile 4	3,579 (19.7%)	1,746 (20.5%)	5,325 (20%)	
Quintile 5	3,650 (20.1%)	1,674 (19.7%)	5,324 (20%)	

Age is shown as mean ± SD; sex as % males. Smoking status, BMI (underweight, normal, overweight, obese), comorbidities (hypertension, diabetes mellitus), family history of CVD, and medication use (lipid-lowering agents) are displayed as % across categories. Marital status is categorized at age 60 years, and wealth index is reported in population quintiles.

BMI = body mass index; CV = cardiovascular; CVD = cardiovascular disease; DANCAVAS = Danish Cardiovascular Screening Trial; MACE = major adverse cardiovascular events; T2DM = type 2 diabetes mellitus.

While exposure levels differed between those who experienced an event and those who did not, the variations were minimal, with median PM_2.5_ levels of 11.87 μg/m^3^ and 11.93 μg/m^3^, respectively, and road traffic noise (L_den_) levels of 50.83 dB and 50.52 dB, respectively (Table 2). Significant correlations were observed between pollutants and noise levels: PM_2.5_ and NO_2_ were strongly correlated (r = 0.84), likely reflecting common sources such as traffic emissions. PM_2.5_ and warm-season O_3_ showed an inverse correlation (r = −0.77), while CO was positively correlated with both PM_2.5_ (r = 0.84) and NO_2_ (r = 0.89). Moderate correlations were observed between road traffic noise (L_den_) and NO_2_ (r = 0.43) and CO (r = 0.42), indicating that areas with higher road traffic noise tend to have elevated levels of traffic-related air pollutants.

**Table 2:** Exposure Summary Table and Spearman Rank Correlation Coefficients Exposure to Air Pollution and Road Traffic Noise Is Shown as Median (IQR) for L_den_ (dB), PM_2.5_, NO_2_, SO_2_, Warm-Season O_3_, and CO (μg/m^3^) as Time-Weighted Averages

Pollutant	Those Who Experienced an Event (n = 4.511)	Those Who Did Not Experience an Event (n = 22,212)	PM_2.5_	NO_2_	Warm-Season O_3_	CO	SO_2_	Road Traffic Noise, L_den_
PM_2.5_, μg/m^3^	11.87 (11.37-12.38)	11.93 (11.40-12.53)	1.00					
NO_2_, μg/m^3^	14.67 (12.87-16,88)	14.46 (12.35-17.01)	0.84	1.00				
Warm-season O_3_, μg/m^3^	54.08 (52.20-55.78)	54.11 (51.92-56.15)	−0.77	−0.96	1.00			
CO, μg/m^3^	193.74 (183.07-209.63)	196.58 (184.70-214.04)	0.84	0.89	−0.89	1.00		
SO_2_, μg/m^3^	6.70 (5.35-8.43)	7.04 (5.55-8.91)	0.73	0.77	−0.78	0.80	1.00	
Road traffic noise, L_den_, dB	50.83 (43.45-58.25)	50.52 (43.24-58.82)	0.32	0.43	−0.40	0.42	0.30	1.00

Exposure measures are quantified from 29 years before each participant’s study inclusion (between 2008 and 2010 for the VIVA cohort and 2014-2018 for the DANCAVAS cohort) until censoring or end of follow up.

CO = carbon monoxide; dB = decibels; Lden = day-evening-night noise level; O_3_ = ozone; NO_2_ = nitrogen dioxide; PM_2.5_ = particulate matter with a diameter <2.5 μm; SO_2_ = sulfur dioxide.

The primary outcome analysis showed that exposure to road traffic noise was associated with a significant increase in the risk of MACE (Table 3). In the fully adjusted model 3, the HR for road traffic noise was 1.075 (95% CI: 1.026-1.128), resulting in a 7.5% increase in risk per 14.9 dB increase in road traffic noise exposure. No significant association was observed between PM_2.5_, NO_2_, SO_2_, CO, or warm-season O_3_ and the composite primary endpoint after full adjustment.

**Table 3:** Cox Proportional Hazards Models to Estimate HRs for the Association Between Pollution Exposure and the Primary Outcome

	Composite Primary Endpoint MACE				Subgroup Analysis	
	Increment (IQR)	Model 1 HR (95% CI)	Model 2 HR (95% CI)	Model 3 HR (95% CI)	Model 3, VIVA Cohort Separately HR (95% CI)	Model 3, DANCAVAS Cohort Separately HR (95% CI)
Road traffic noise, L_den_	14.9 dB	**1.096 (1.046-1.148)**	**1.076 (1.027-1.128)**	**1.075 (1.026-1.128)**	**1.080 (1.026-1.137)**	1.013 (0.875-1.172)
PM_2.5_	1.02 μg/m^3^	0.997 (0.958-1.037)	0.996 (0.957-1.037)	1.001 (0.961-1.043)	0.998 (0.950-1.049)	0.992 (0.883-1.115)
NO_2_	4.09 μg/m^3^	1.020 (0.985-1.056)	1.017 (0.982-1.053)	1.023 (0.988-1.059)	1.024 (0.981-1.068)	0.956 (0.841-1.086)
Warm-season O_3_	3.65 μg/m^3^	0.980 (0.951-1.009)	0.981 (0.952-1.010)	0.976 (0.947-1.005)	0.976 (0.941-1.013)	1.030 (0.923-1.151)
CO	26.84 μg/m^3^	**1.029 (1.001**-**1.057)**	1.022 (0.994-1.050)	1.025 (0.997-1.053)	1.025 (0.994-1.057)	0.977 (0.881-1.083)
SO_2_	3.12 μg/m^3^	1.007 (0.994-1.020)	1.005 (0.992-1.018)	1.005 (0.992-1.018)	1.006 (0.992-1.020)	0.962 (0.891-1.039)

Time of baseline defines the onset of risk time, and exit is time of event or censoring. HRs for the primary composite endpoint of MACE (nonfatal myocardial infarction, nonfatal stroke, cardiovascular mortality, and revascularization procedures [CABG, PCI, PTA, leg bypass surgery]) per IQR increase in pollutant levels (noise, PM_2.5_, NO_2_, warm-season O_3_, CO, SO_2_). The table shows HRs adjusted across 3 models: model 1 adjusts for baseline inclusion year; model 2 adds adjustments for lifestyle factors (smoking status, BMI), and family history of CVD; model 3 further adjusts for marital status and household wealth index.

Abbreviations as in Tables 1 and 2.

**Figure 1:**
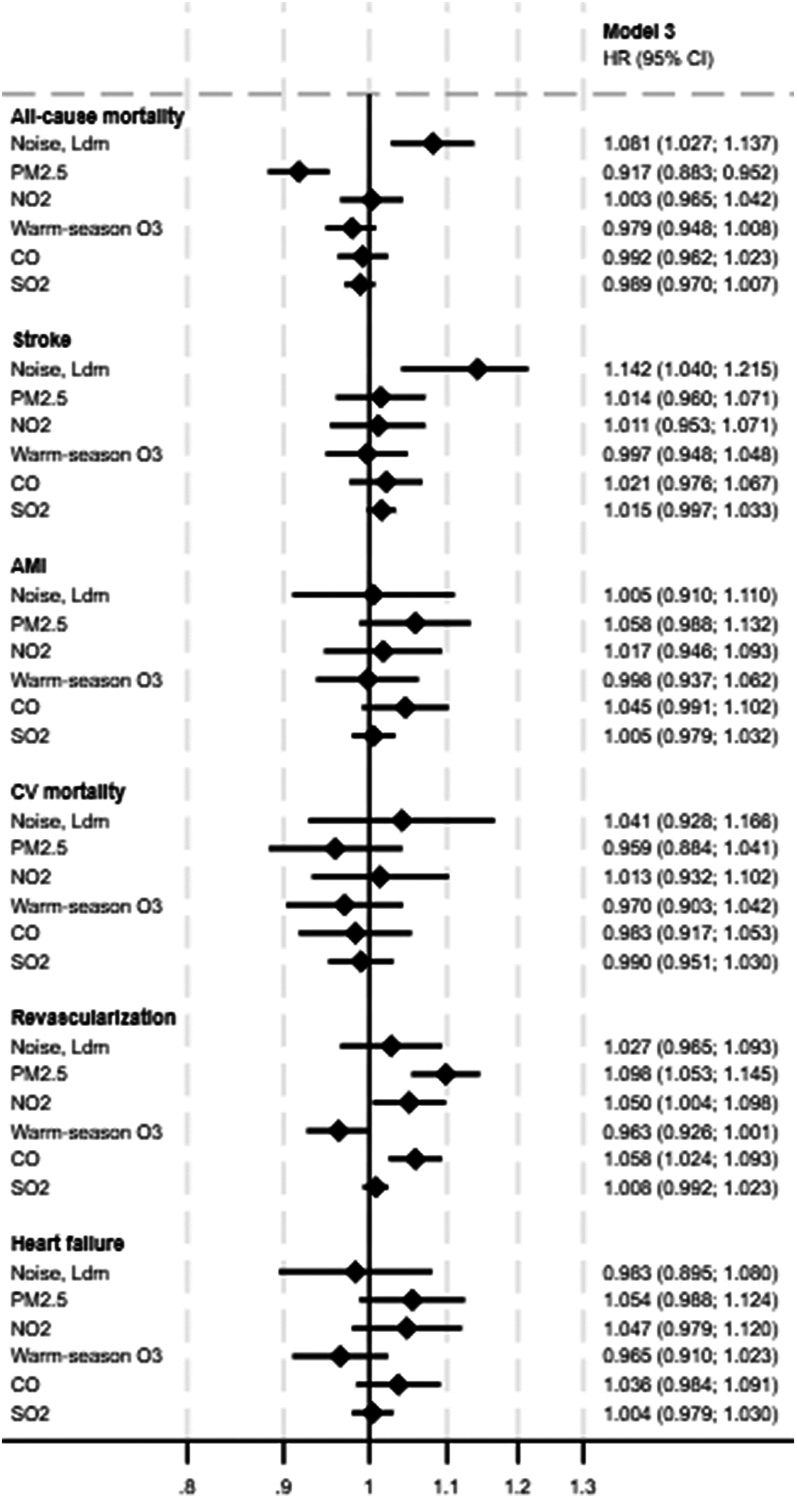
**HRs for Secondary Cardiovascular Outcomes in Model 3** HRs and 95% CI for secondary stroke, myocardial infarction, cardiovascular mortality, revascularization, heart failure, and all-cause mortality. The results are from model 3, which is fully adjusted for baseline inclusion year, smoking status, body mass index, family history of cardiovascular disease, and socioeconomic factors. HRs are shown for PM_2.5_, NO_2_, CO, SO_2_, warm-season O_3_, and road traffic noise (L_den_). CO = carbon monoxide; CV = cardiovascular; Ldm = day-evening-night noise level; NO_2_ = nitrogen dioxide; O_3_ = ozone; PM_2.5_ = particulate matter with a diameter <2.5 μm; SO_2_ = sulfur dioxide.

The secondary outcomes analysis provided additional insights into these associations (Table 4 and Figure 1). Stroke was the most positively associated outcome, with road traffic noise showing a significant association (HR: 1.124; 95% CI: 1.040-1.215) in the fully adjusted model 3. For AMI, no significant association was found with road traffic noise (HR: 1.005; 95% CI: 0.910-1.110) or any of the other pollutants. Similarly, CV mortality did not show any significant association with the pollutants, including road traffic noise (HR: 1.041; 95% CI: 0.928-1.166), indicating no clear link. Revascularization procedures were positively associated with PM_2.5_, NO_2_, and CO after full adjustment (HR: 1.098; 95% CI: 1.053-1.145; HR: 1.050; 95% CI: 1.004-1.098; and HR: 1.058; 95% CI: 1.024-1.093, respectively). Heart failure showed no significant association with any exposure. All-cause mortality, which included both CV and non-CV deaths, was significantly associated with road traffic noise exposure. The fully adjusted model showed an 8.1% increase in risk per 14.9 dB increase in road traffic noise (HR: 1.081; 95% CI: 1.027-1.137), suggesting that noise pollution has a broader impact on overall mortality beyond CV-specific outcomes. No significant associations were observed for PM_2.5_ or other pollutants with all-cause mortality.

**Table 4:** Cox Proportional Hazards Models to Estimate HRs for the Association Between Pollution Exposure and Secondary CVD Outcomes

	No. of Events	Model 1	Model 2	Model 3
All-cause mortality	3,821			
Noise, L_den_		1.121 (1.066-1.178)	1.098 (1.044-1.155)	1.081 (1.027-1.137)
PM_2.5_		0.910 (0.878-0.944)	0.909 (0.876-0.943)	0.917 (0.883-0.952)
NO_2_		0.996 (0.959-1.034)	0.994 (0.957-1.032)	1.003 (0.965-1.042)
Warm-season O_3_		0.989 (0.958-1.022)	0.988 (0.957-1.021)	0.979 (0.948-1.012)
CO		0.993 (0.963-1.024)	0.989 (0.959-1.020)	0.992 (0.962-1.023)
SO_2_		0.989 (0.971-1.009)	0.987 (0.968-1.007)	0.989 (0.970-1.007)
Stroke	1,624			
Noise, L_den_		1.148 (1.063-1.239)	1.129 (1.045-1.220)	1.124 (1.040-1.215)
PM_2.5_		1.006 (0.954-1.061)	1.003 (0.950-1.059)	1.014 (0.960-1.071)
NO_2_		1.005 (0.949-1.065)	1.002 (0.946-1.062)	1.011 (0.953-1.071)
Warm-season O_3_		1.003 (0.955-1.054)	1.003 (0.954-1.055)	0.997 (0.948-1.048)
CO		1.025 (0.981-1.071)	1.017 (0.973-1.063)	1.021 (0.976-1.067)
SO_2_		1.016 (0.997-1.034)	1.015 (0.996-1.033)	1.015 (0.997-1.033)
AMI	1,059			
Noise, L_den_		1.020 (0.925-1.124)	1.000 (0.906-1.105)	1.005 (0.910-1.110)
PM_2.5_		1.042 (0.975-1.113)	1.050 (0.982-1.123)	1.058 (0.988-1.132)
NO_2_		1.003 (0.934-1.077)	1.007 (0.937-1.082)	1.017 (0.946-1.093)
Warm-season O_3_		1.009 (0.950-1.073)	1.006 (0.946-1.071)	0.998 (0.937-1.062)
CO		1.043 (0.990-1.099)	1.039 (0.985-1.095)	1.045 (0.991-1.102)
SO_2_		1.005 (0.978-1.033)	1.005 (0.979-1.032)	1.005 (0.979-1.032)
CV mortality	787			
Noise, L_den_		1.109 (0.992-1.239)	1.069 (0.955-1.197)	1.041 (0.928-1.166)
PM_2.5_		0.937 (0.865-1.015)	1.939 (0.866-1.018)	0.959 (0.884-1.041)
NO_2_		0.991 (0.912-1.077)	0.992 (0.912-1.079)	1,013 (0.932-1.102)
Warm-season O_3_		0.993 (0.925-1.067)	0.990 (0.921-1.064)	0.970 (0.903-1.042)
CO		0.976 (0.910-1.047)	0.974 (0.908-1.045)	0.983 (0.917-1.053)
SO_2_		0.989 (0.947-1.032)	0.987 (0.946-1.030)	0.990 (0.951-1.030)
Revascularization	2,676			
Noise, L_den_		1.039 (0.978-1.104)	1.021 (0.960-1.086)	1.027 (0.965-1.093)
PM_2.5_		**1.090 (1.046-1.136)**	**1.095 (1.051-1.141)**	**1.098 (1.053-1.145)**
NO_2_		**1.045 (1.000-1.092)**	1.044 (0.999-1.091)	**1.050 (1.004-1.098)**
Warm-season O_3_		0.967 (0.931-1.005)	0.968 (0.931-1.006)	0.963 (0.926-1.001)
CO		**1.057 (1.023-1.091)**	**1.053 (1.020-1.088)**	**1.058 (1.024-1.093)**
SO_2_		1.011 (0.995-1.026)	1.008 (0.992-1.023)	1.008 (0.992-1.023)
Heart failure	1,178			
Noise, L_den_		1.009 (0.920-1.107)	0.987 (0.899-1.084)	0.983 (0.895-1.080)
PM_2.5_		1.019 (0.957-1.086)	1.028 (0.965-1.096)	1.054 (0.988-1.124)
NO_2_		1.024 (0.957-1.094)	1.027 (0.960-1.098)	1.047 (0.979-1.120)
Warm-season O_3_		0.982 (0.927-1.041)	0.980 (0.924-1.038)	0.965 (0.910-1.023)
CO		1.037 (0.986-1.091)	1.036 (0.984-1.091)	1.047 (0.996-1.102)
SO_2_		1.005 (0.978-1.032)	1.003 (0.977-1.030)	1.004 (0.979-1.030)

Time of baseline defines the onset of risk time, and exit is time of event or censoring. HRs for the secondary outcomes (all-cause mortality, stroke, AMI, CV mortality, revascularization, heart failure) per IQR increase in pollutant levels (road traffic noise, PM_2.5_, NO_2_, warm-season O_3_, CO, SO_2_). The table shows HRs adjusted across 3 models: model 1 adjusts for baseline inclusion year; model 2 adds adjustments for lifestyle factors (smoking status, BMI), and family history of CVD; model 3 further adjusts for marital status and household wealth index.

AMI = acute myocardial infarction; other abbreviations as in Tables 1 and 2.

When the 2 cohorts were analyzed separately, we found notable differences in follow-up time and event rates. The VIVA cohort had a significantly longer follow-up period (8.3 years vs 3.3 years in DANCAVAS) and experienced 4,115 total events compared to 396 events in the DANCAVAS cohort. In separate analyses (Supplemental Table 1), road traffic noise was significantly associated with all-cause mortality (HR: 1.100; 95% CI: 1.041-1.161) and stroke (HR: 1.135; 95% CI: 1.043-1.234) in the VIVA cohort. Additionally, PM_2.5_ exposure was significantly associated with revascularization (HR: 1.114; 95% CI: 1.060-1.171).

**Figure 2:**
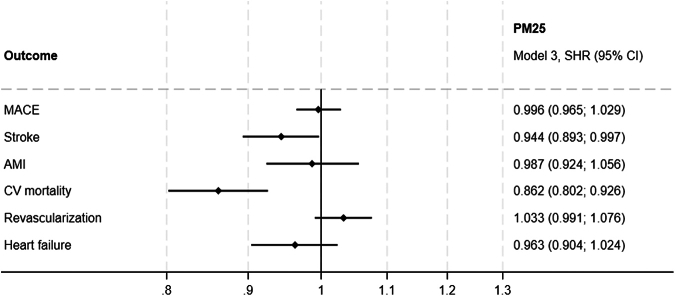
**Fine and Gray Competing Risk Analysis for PM_2.5_** Subdistribution HRs and 95% CIs from the Fine and Gray competing risk model for PM_2.5_ exposure. The competing risk model accounts for the potential influence of noncardiovascular mortality when estimating the association between long-term PM_2.5_ exposure and cardiovascular outcomes. Subdistribution HRs are shown for PM_2.5_ exposure in relation to major adverse cardiovascular events, stroke, myocardial infarction, cardiovascular mortality, revascularization, and heart failure. MACE = major adverse cardiovascular events; other abbreviations as in Figure 1.

**Figure 3:**
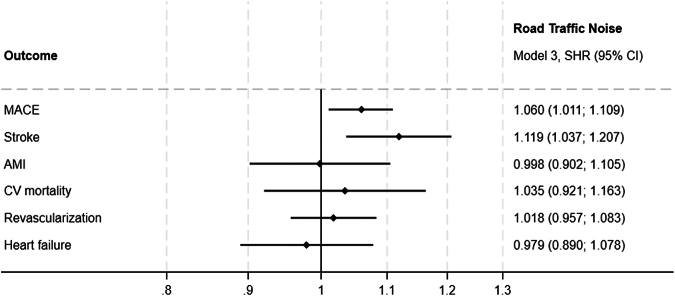
**Fine and Gray Competing Risk Analysis for Road Traffic Noise** Subdistribution HRs and 95% CIs from the Fine and Gray competing risk model for road traffic noise exposure. The competing risk model accounts for the potential influence of noncardiovascular mortality when estimating the association between road traffic noise and cardiovascular outcomes. Subdistribution HRs are shown for road traffic noise in relation to major adverse cardiovascular events, stroke, myocardial infarction, cardiovascular mortality, revascularization, and heart failure. SHR = subdistribution HR; other abbreviations as in Figures 1 and 2.

To account for competing risks, we conducted a Fine and Gray analysis, treating all-cause mortality as a competing event for outcomes that did not include CV mortality (Table 5, Figures 2 and 3). This analysis confirmed the association between road traffic noise and increased risk of stroke, with a subdistribution HR (SHR) of 1.119 (95% CI: 1.037-1.207), supporting the findings of the primary analysis. For MACE, road traffic noise exposure remained significantly associated with an increased risk (SHR: 1.060; 95% CI: 1.011-1.111), while PM_2.5_ exposure did not show a significant association (SHR: 0.976; 95% CI: 0.965-1.029).

**Table 5:** Fine and Gray Competing Risk Analysis

	No. of Events	SHR Model 1, Road Traffic Noise (95% CI)	SHR Model 1, PM_2.5_ (95% CI)	SHR Model 2, Road Traffic Noise (95% CI)	SHR Model 2, PM_2.5_ (95% CI)	SHR Model 3, Road Traffic Noise (95% CI)	SHR Model 3, PM_2.5_ (95% CI)
MACE	4,505	**1.076 (1.028-1.127)**	0.996 (0.966-1.028)	**1.057 (1.009-1.108)**	0.993 (0.962-1.025)	**1.060 (1.011-1.111)**	0.996 (0.965-1.029)
Stroke	1,624	**1.134 (1.053-1.222)**	**0.943 (0.893-0.995)**	**1.120 (1.038-1.207)**	**0.937 (0.888-0.990)**	**1.119 (1.037-1.207)**	**0.944 (0.893-0.997)**
AMI	1,059	1.007 (0.912-1.112)	0.980 (0.918-1.046)	0.990 (0.895-1.095)	0.984 (0.922-1.050)	0.998 (0.902-1.105)	0.987 (0.924-1.056)
CV mortality	787	1.095 (0.978-1.227)	0.857 (0.799-0.919)	1.060 (0.944-1.190)	0.853 (0.794-0.915)	1.035 (0.921-1.163)	0.862 (0.802-0.926)
Revascularization	2,676	1.025 (0.966-1.089)	1.031 (0.991-1.073)	1.009 (0.949-1.072)	1.032 (0.992-1.074)	1.018 (0.957-1.083)	1.033 (0.991-1.076)
Heart failure	1,178	0.997 (0.908-1.095)	0.942 (0.886-1.002)	0.979 (0.891-1.077)	0.945 (0.889-1.005)	0.979 (0.890-1.078)	0.963 (0.904-1.024)

Fine and Gray competing risk analysis, subdistribution HRs for primary and secondary outcomes, with death from noncardiovascular causes treated as the competing risk. The analysis shows the impact of PM_2.5_ and noise pollution as the explanatory variables. Adjustments are made across 3 models to account for confounding factors: model 1 adjusts for baseline inclusion year; model 2 adds adjustments for lifestyle factors (smoking status, BMI), and family history of CVD; model 3 further adjusts for marital status and household wealth index.

SHR = subdistribution HR; other abbreviations as in Tables 1 and 4.

**Figure 4:**
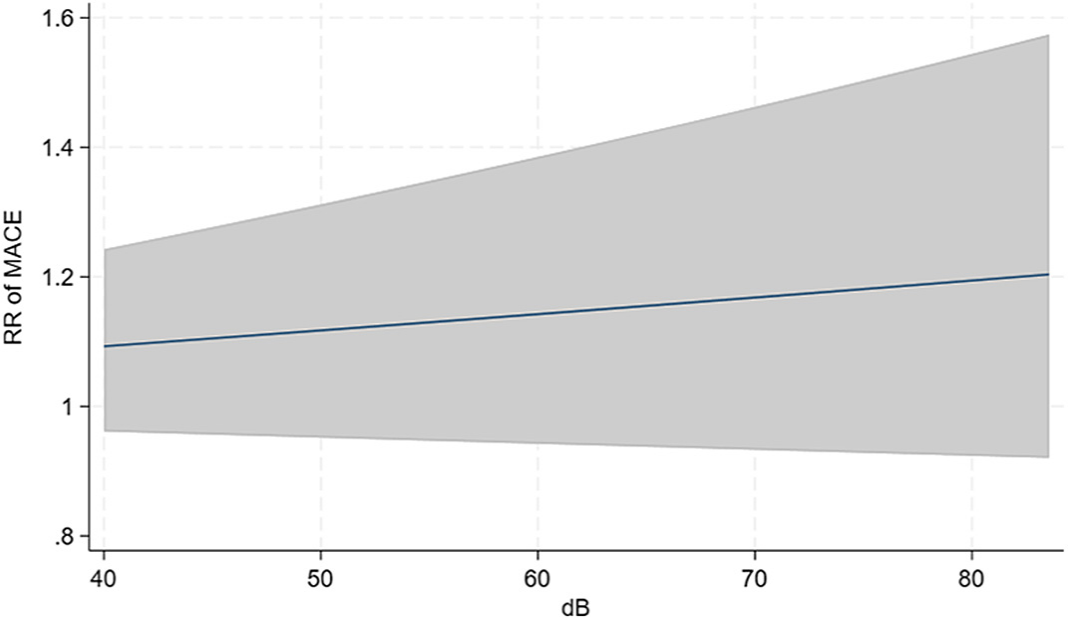
**Dose-Response Curve of Road Traffic Noise and Cardiovascular Risk** Dose-response relationship between road traffic noise (L_den_, dB) and major adverse cardiovascular events. The solid line represents relative risk estimates, with the shaded area indicating the 95% CI, based on Cox proportional hazards models. Association between residential road traffic noise exposure and the relative risk of MACE. The figure shows the dose-response relationship between residential road traffic noise exposure (L_den_, in decibels [dB]) and the relative risk of major adverse cardiovascular events. dB = decibels; Lden = day-evening-night noise level; RR = relative risk; other abbreviation as in Figures 1 and 2.

**Figure 5:**
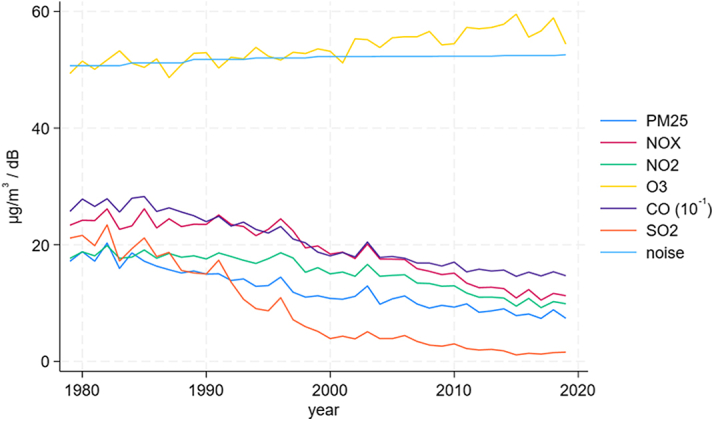
T**emporal Trends in Air Pollution and Road Traffic Noise** Temporal trends in mean concentrations of PM_2.5_, NO_2_, O_3_, CO (scaled by 10^−1^), SO_2_, and road traffic noise (L_den_). Air pollution levels declined over time, while noise and ozone remained stable. The temporal trends from 1979 to 2019 in mean concentrations of air pollutants—PM_2.5_, NO_2_, O_3_, CO (scaled by 10^−1^), and SO_2_—and road traffic noise. The graph shows a decline in air pollutant levels over the past 4 decades, while ozone and road traffic noise levels have remained relatively stable. NO_x_ = nitrogen oxides; other abbreviations as in Figures 1 and 4.

The dose-response analysis found no clear relationship between increasing road traffic noise levels and the risk of MACE (Figure 4). Over the study period (1979-2019), PM_2.5_, NO_2_, CO, and SO_2_ levels significantly declined, reflecting improved air quality, whereas road traffic noise levels remained stable with minimal fluctuations (Figure 5). This stability in noise exposure, despite reductions in air pollutants, indicates that road traffic noise continues to be a consistent environmental stressor affecting CV health independently. Warm-season O_3_ levels, however, remained constant or showed a slight increase.

## Discussion

This study found that long-term exposure to road traffic noise was significantly associated with an increased risk of MACE with a 14.9 dB increase in noise levels leading to a 7.5% higher risk of MACE (Central Illustration). These findings align with those of previous studies showing that road traffic noise contributes to increased CVD risk and all-cause mortality.^12^^,^^42^^,^^43^ Other studies have shown that road traffic noise significantly increases the risk of myocardial infarction, stroke, and other CV outcomes, independent of air pollution exposure.^13^^,^^42^^,^^43^ A nationwide cohort study in Switzerland found that adjusting for air pollutants such as NO_2_ and PM_2.5_ did not substantially alter the HRs for road traffic noise-related myocardial infarction mortality, but adjusting for noise reduced the HRs for air pollutants.^42^ A lack of significant associations between air pollutants and CVD, other than revascularization procedures, in our pooled cohort contrasts with studies linking PM_2.5_ and NO_2_ to CVD.^10^^,^^11^^,^^44^^,^^45^

**Central Illustration:**
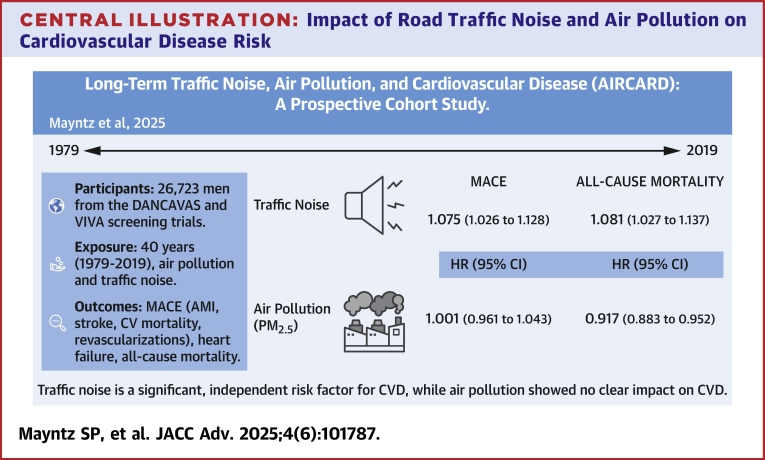
**Impact of Road Traffic Noise and Air Pollution on Cardiovascular Disease Risk** AIRCARD study findings on road traffic noise and air pollutants and their association with cardiovascular events (MACE) among men aged 65 to 74 years. HRs per IQR increase are fully adjusted. Road traffic noise significantly increased MACE and all-cause mortality risk; PM_2.5_ showed no increased associations at low exposure levels. Findings highlight road traffic noise as an important factor for cardiovascular risk in populations with relatively low air pollution exposure. AIRCARD = AIR pollution and CARDiovascular disease; AMI = acute myocardial infarction; CVD = cardiovascular disease; DANCAVAS = Danish Cardiovascular Screening Trial; other abbreviations as in Figure 1.

The relatively low air pollution levels in Denmark, likely because of strict environmental regulations, may explain the absence of significant association between air pollutants and CV outcomes, as exposure levels may be below the threshold for adverse CVD effects. This could be supported by the findings of Hvidtfeldt et al (2019), who investigated the association between long-term exposure to PM_2.5_ and CVD mortality in Denmark.^44^ They reported a HR of 1.29 (95% CI: 1.13-1.47) per 5 μg/m^3^ increase in PM_2.5_, and a HR of 1.24 (95% CI: 1.06-1.45) when including adjustments for road traffic noise. Their study population was based in the capital and the second largest city, where the mean PM_2.5_ levels were above 18 μg/m^3^, which was significantly higher than the mean level of approximately 12 μg/m^3^ in our study (and <8 μg/m^3^ in the last 10 years of study). This suggests that the harmful CV effects of PM_2.5_ may become more pronounced at higher exposure levels, and there may be a lower threshold below which the association is not detectable, contrary to current evidence.^46^^,^^47^

Our study likely underestimates the true CV risk due to early interventions given to participants for subclinical CVD during screening, particularly in the DANCAVAS cohort, where treatments like statins, aspirin, and anticoagulants were more commonly administered.^27^ In contrast, 3.3% of the participants in the VIVA cohort received such treatments, suggesting that the underestimation effect may be less pronounced in this group. Consequently, our results do not fully reflect the natural history of CVD in untreated populations, in which the absence of screening and early intervention would likely result in higher rates of adverse outcomes. Nevertheless, even with these interventions, we still observed significant associations, indicating that the risks posed by these exposures are not fully mitigated by the treatments initiated.

The significantly longer follow-up time in the VIVA cohort (8.3 years vs 3.3 years) and the higher number of events (4,115 vs 396) likely explain the stronger associations observed in VIVA for PM_2.5_ and outcomes such as stroke, AMI, and heart failure. Another possibility is that approximately 10 times more participants in DANCAVAS received preventive treatment than in VIVA, which may have reduced the incidence of CV events in DANCAVAS and limited the ability to detect significant associations in the pooled cohort. If the study had been conducted in a cohort without screening, early detection, and intervention, such as a general population cohort, we might have observed stronger and significant associations between PM_2.5_ exposure and CV outcomes.

The dose-response analysis found a nonsignificant relationship between increasing levels of road traffic noise and relative risk of MACE (Figure 4). Over the study period, PM_2.5_, NO_2_, CO, and SO_2_ levels significantly declined, reflecting improved air quality, whereas road traffic noise levels remained stable with minimal fluctuations (Figure 5). This stability in noise exposure, despite reductions in air pollutants, indicates that road traffic noise continues to be a consistent environmental factor that potentially affects CV health independently. These observations could suggest that the ongoing burden of CVD may be more attributable to persistent road traffic noise exposure rather than air pollution during long-term exposure to low-level ambient air pollution. As air quality has improved, one might expect a corresponding decrease in CVD incidence if air pollution was a primary driver of CV risk in a population like the one we investigated, as proposed by the State of Global Air Report 2024, ranking air pollution the second largest risk factor of deaths in 2021, globally.^3^ However, the lack of decline in CVD rates, coupled with stable noise exposure levels and decreased levels of air pollution, supports the idea that road traffic noise may play a larger role in sustaining the CVD burden in populations with relatively low-level ambient air pollution. However, while our findings show a decline in ambient air pollutants over time, it is important to note that the benefits of improved air quality may be partly offset by the increasing aging population in HICs. As people live longer, their cumulative exposure to pollutants increases, and age-related physiological changes may heighten susceptibility to CV effects. This means that even with lower or stable ambient levels, the overall burden of air pollution on CV health may remain substantial. This aspect stresses the need for efforts to reduce environmental exposures, particularly among vulnerable older populations.

Our findings suggest that studies that do not consider noise exposure may overestimate the CV burden attributed to air pollution.^42^^,^^48^^,^^49^ Additionally, although both noise and air pollution share some biological pathways, such as oxidative stress and inflammation, the mechanisms through which noise impacts CV health—particularly through sleep disruption and stress hormone release—appear to be more potent in driving CV risk.^48^^,^^50^ Our study adds to the growing body of evidence that transportation noise is a critical and independent risk factor for CVD.

A key strength of our study is its prospective cohort design, which allowed for the collection and analysis of long-term data on both air pollution and road traffic noise exposure over a period of up to 4 decades. This exposure window provides an opportunity to evaluate the chronic effects of environmental factors on CV outcomes. In addition, the use of high-precision exposure models for both air pollution and noise provide an assessment of individual residential exposure levels. Modeling road traffic noise at the most exposed façade and air pollutants at a fine spatial resolution helped minimize exposure misclassification. Our study also benefited from the use of individual-level covariates obtained from the 2 screening trials and national registries. The availability of these covariates enabled us to adjust for major confounders that could influence CV risk, ensuring that the observed associations between environmental exposures and CV events were not confounded by lifestyle or demographic factors. This level of detailed covariate adjustment is often lacking in studies that rely solely on registry data.^47^^,^^51^, ^52^, ^53^ The large sample size and long follow-up period further supported the statistical power to detect meaningful associations. A key strength of this study is the pooling of data from the highly comparable DANCAVAS and VIVA cohorts, which were designed by the same investigators and recruited from similar geographical areas. Given Denmark’s uniform population and participant mobility, adjusting for baseline inclusion year accounted for differences in enrollment periods and follow-up times. Additionally, some participants were enrolled in both trials, and this overlap was carefully handled. Further adjusting for study site could risk overadjustment without adding meaningful distinctions between the cohorts.

Despite the strengths of this study, several limitations should be acknowledged. First, we lacked data on important lifestyle factors such as alcohol consumption, dietary habits, and access to residential greenspace. The absence of these variables may have introduced residual confounding. Also, like in existing literature, only baseline confounders were adjusted for, without accounting for time-varying factors like lifestyle or socioeconomic changes during follow-up. Participants' risk profiles may change over time.^54^

Second, our data on smoking were limited, with no information available on smoking duration or intensity. While we adjusted for smoking status, the lack of detailed smoking history may have affected the precision of our estimates. Also, we assessed ambient environmental exposures solely based on participants' residential addresses, without accounting for time spent at other locations, such as workplaces. This may have led to some misclassification of exposure, especially for individuals who spend a significant portion of their day away from home.

Third, our cohort consisted entirely of men, leading to potential gender bias. CV responses to environmental exposures may differ between men and women, limiting the generalizability of our findings to the broader population. Additionally, although participants were randomly invited, those who chose to participate may represent a healthier subset of the population, which, along with the preventive interventions they received, could lead to an underestimation of the actual CVD incidence and mortality rates.

Our findings have important implications for public health and policy. They emphasize the need to consider road traffic noise as a significant environmental health hazard that contributes to CVD risk. Policy interventions aimed at reducing road traffic noise exposure—such as urban planning revisions to reduce traffic density near residential areas, implementation of noise barriers, enforcement of stricter noise regulations, and promotion of quieter vehicle technologies—could have meaningful impacts on reducing CVD risk at the population level, where air pollution levels are already low (PM_2.5_ annual levels at or below 8-12 μg/m^3^). Balancing environmental health efforts between air pollution and noise pollution mitigation is crucial, as both factors affect public health, but may require different strategies and resource allocations.

Future research should aim to include more diverse and inclusive cohorts involving women and different age groups, to improve the generalizability of the findings. Incorporating comprehensive exposure assessments that account for time spent away from home, using personal exposure monitors, or including workplace exposure data would provide a more accurate assessment of total environmental exposure. Longitudinal studies with time-varying covariates could account for changes in participants’ lifestyles and socioeconomic status over time, reducing potential residual confounding. Intervention studies testing the effectiveness of noise reduction strategies on CV outcomes would provide valuable insights into causal relationships and potential public health benefits. It is also important to consider the potential synergistic effects of combined exposure to air pollution and noise. Some studies suggest that concurrently elevated exposure to both factors may have additive or multiplicative effects on CV risk.^23^^,^^55^ Future research should explore these interactions by using integrated exposure models to fully understand their joint impact on CV health. Also, while our study focused on estimating the independent effects of air pollution and road traffic noise on CV outcomes, mediation through hypertension, T2DM, and hypercholesterolemia remains an important area for future research. Given their role as intermediates in the causal pathway, dedicated mediation analyses could provide further insights but were beyond the scope of our current study.

## Conclusions

Our findings highlight the role of road traffic noise as a significant, independent risk factor for CVD. This study provides evidence linking long-term exposure to road traffic noise with an increased risk of MACE. Although air pollution remains a critical concern globally, road traffic noise should be recognized as a distinct contributor to CV risk in populations exposed to low-level ambient air pollution. The results of this study suggest that omitting noise exposure may lead to an overestimation of the CV burden attributed solely to air pollution.

## Funding support and author disclosures

This work was supported by the Region of Southern Denmark (Odense, Denmark) and by Shipowner Per Henriksen’s Foundation (Copenhagen, Denmark). The sponsors were not involved in the project. The authors have reported that they have no relationships relevant to the contents of this paper to disclose.

Perspectives **COMPETENCY IN MEDICAL KNOWLEDGE:** Clinicians should recognize road traffic noise as a significant environmental contributor to CVD risk, independently from air pollutants, especially among older populations.

**TRANSLATIONAL OUTLOOK:** Further research should explore targeted noise-reduction interventions and assess their effectiveness in lowering CVD incidence, emphasizing the need for incorporating noise assessments into clinical and public health guidelines.
