# Supplementary material for: Long-Term Road Traffic Noise, Air Pollution, and Cardiovascular Disease AIRCARD: A Prospective Cohort Study
Source: JACC Adv. 2025 May 21;4(6):101787. doi: 10.1016/j.jacadv.2025.101787 (PMC12150041; doi:10.1016/j.jacadv.2025.101787)
Supplement: Supplemental Data [file mmc1.docx]

# SUPPLEMENTAL APPENDIX

# Peripheral revascularization procedure codes

The following codes were used to capture peripheral revascularization procedures. The codes are from the NOMESCO Classification of Surgical Procedures related to vascular and endovascular interventions derived from the Danish national coding system for medical procedures.

**Peripheral Reconstructions**

PAH25: Bypass from carotid to subclavian artery

PAH30: Bypass from subclavian artery

PBH10: Bypass from axillary artery

PBH20: Bypass from brachial artery

PBH99: Bypass from another artery in the upper extremity (radial/ulnar)

PGH23: Axillo-bifemoral bypass

PGH22: Axillo-femoral bypass

PCH10: Bypass from the abdominal aorta (supraceliac-juxtarenal)

PCH20: Bypass from the celiac trunk

PCH30: Bypass from the superior mesenteric artery

PCH40: Bypass from the renal artery

PCH99: Bypass from another visceral artery (inferior mesenteric)

PDH20: Aorto-iliac bypass

PDH21: Aorto-biiliac bypass

PDH22: Aorto-iliac/femoral bypass, contralateral

PDH23: Aorto-femoral bypass

PDH24: Aorto-bifemoral bypass

PDH30: Iliac bypass

PDH35: Iliac-femoral bypass

PGH40: Femoro-femoral bypass

PEH20: Bypass from femoral to popliteal artery above the knee

PEH30: Bypass from femoral to popliteal artery below the knee

PFH10: Bypass from popliteal to popliteal artery

PFH23: Bypass from femoral/popliteal to tibiofibular trunk

PFH24: Bypass from femoral/popliteal to proximal posterior tibial artery

PFH25: Bypass from femoral/popliteal to distal posterior tibial artery

PFH29: Bypass from femoral/popliteal to plantar artery of the foot

PFH26: Bypass from femoral/popliteal to proximal peroneal artery

PFH27: Bypass from femoral/popliteal to distal peroneal artery

PFH21: Bypass from femoral/popliteal to proximal anterior tibial artery

PFH22: Bypass from femoral/popliteal to distal anterior tibial artery

PFH28: Bypass from femoral/popliteal to dorsalis pedis artery

PAF20: Thrombendarterectomy of the common carotid artery

PAF21: Thrombendarterectomy of the internal carotid artery

PAF22: Thrombendarterectomy of the external carotid artery

PAF30: Thrombendarterectomy of the subclavian artery

PDF10: Thrombendarterectomy of the infrarenal aorta

PDF15: Thrombendarterectomy of the aorto-iliac region

PDF30: Thrombendarterectomy of the iliac artery

PDF35: Thrombendarterectomy of the iliac-femoral region

PEF10: Thrombendarterectomy of the common femoral artery

PEF11: Thrombendarterectomy of the deep femoral artery

PEF12: Thrombendarterectomy of the superficial femoral artery

**Endovascular Reconstructions**

PAP30: Percutaneous transluminal angioplasty (PTA) of the subclavian artery

PCP40: PTA of the renal artery

PCP99: PTA of another visceral artery

PDP10: PTA of the infrarenal aorta

PDP30: PTA of the iliac artery

PEP10: PTA of the common femoral artery

PEP11: PTA of the deep femoral artery

PEP12: PTA of the superficial femoral artery

PFP10: PTA of the popliteal artery

PFP30: PTA of the crural or foot artery

PBU83: PTA of an arteriovenous fistula in the upper extremity

PCU83: PTA of a bypass on the suprarenal aorta and visceral arteries

PDU83: PTA of a bypass from the infrarenal aorta and iliac artery

PEU83: PTA of a bypass from the femoral to the popliteal artery above or below the knee

PEU83: PTA of a bypass from the femoral artery and its branches

PFU83: PTA of a bypass from the femoral/popliteal/crural arteries

**Aneurysm Operations**

PDG10: Aneurysm surgery on the infrarenal aorta

PDG20: Aneurysm surgery with aorto-iliac bypass

PDG21: Aneurysm surgery with aorto-biiliac bypass

PDG22: Aneurysm surgery with aorto-iliac/femoral bypass, contralateral

PDG23: Aneurysm surgery with aorto-femoral bypass

PDG24: Aneurysm surgery with aorto-bifemoral bypass

PDG30: Aneurysm surgery on the iliac artery

PDG35: Aneurysm surgery with iliac-femoral bypass

PDG99: Aneurysm surgery on the iliac artery with another type of bypass

KPDQ10: Insertion of an endoprosthesis in the infrarenal aorta

KPDQ20: Insertion of an endoprosthesis in the aortoiliac region

KPDQ21: Insertion of an endoprosthesis in the aortobiiliac region

KPDQ30: Insertion of an endoprosthesis in the iliac artery

# Confounders, mediators, and moderators

We have included the following covariates for both cohorts: age, smoking status, BMI, hypertension, T2DM, lipid-lowering agent use, family history of CVD, marital status, household wealth index, educational level, and occupational status.

Age, smoking status, BMI, family history of CVD, and socioeconomic status (measured by marital status and household wealth index) are considered confounders. These variables are well-established risk factors for CVD and may influence both exposure to pollution and the outcomes of interest. Adjusting for these confounders helps to isolate the effect of air and noise pollution on CVD outcomes.

Hypertension, T2DM, and hypercholesterolemia are treated as mediators in our analysis. These conditions are often intermediate steps in the causal pathway from pollution exposure to CVD (1-3). By not adjusting for these variables in the primary models, we aim to capture the total effect of pollution, including its impact through these mediating pathways.

Educational level and occupational status are not adjusted for in our models due to the characteristics of our study population. The educational level is almost uniform across the elderly population in our cohorts, minimizing its variability and potential confounding effect. Similarly, the majority of our participants are retired, making occupational status a non-contributory variable to our analysis.

**Supplemental Figure 1.** Directed Acyclic Graph of covariates and exposures


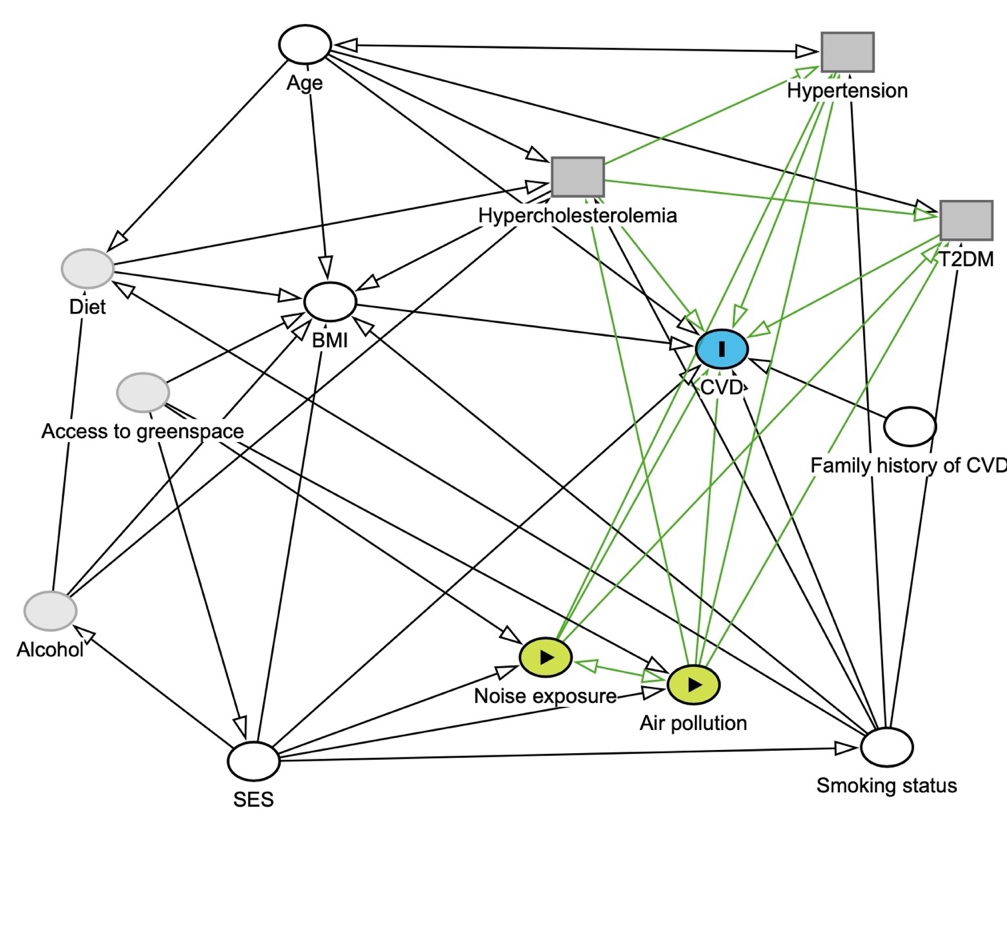


*Figure legend: DAG of the relationships between covariates, exposures, and the CVD outcome. The colors and shapes represent categories of variables: Blue (oval) represents the CVD outcome; green (oval) represents the exposures (air pollution, noise exposure); white (round) indicates confounders that are adjusted for in the analysis (age, BMI, smoking status, family history of CVD, socioeconomic status - SES); grey (square) indicates mediators that are not adjusted for to capture their mediating effects (hypertension, T2DM, hypercholesterolemia); and light grey/transparent grey (round) shows confounders that are not adjusted for due to lack of access to these variables (diet, alcohol, access to greenspace). Arrows indicate the direction of influence or association, with arrows pointing from causes to effects. Confounders are variables that are controlled for in the analysis to reduce bias in estimating the effects of exposures on the outcome. Mediators are on the causal pathway between the exposures and the outcome, representing intermediate steps through which the exposures might affect the outcome. Unavailable confounders could influence the outcome but are not included due to the unavailability of data.*

#

# Statistical Analysis Plan changes after publication

Following the publication of the Statistical Analysis Plan for the AIRCARD study, few modifications were made. In the initial SAP, hypertension, T2DM, and hypercholesterolemia were treated as confounders. Upon further reflection, these variables were reclassified as mediators in the analyses. This change is based on the understanding that these factors lie on the causal pathway between pollution exposure and CVD, and thus should not be adjusted as confounders. Also, the manuscript revises the adjustment strategy outlined in the SAP by excluding educational level and occupational status due to their uniformity within the study population and the predominantly retired status of participants. Instead, the analysis in the manuscript focuses on more pertinent socioeconomic factors such as the household wealth index and marital status, which are considered in the fully adjusted models to better account for potential confounding.

We decided not to implement the FDR correction and hierarchical testing. It was based on several considerations. First, the exploratory nature of the AIRCARD study, focusing on hypothesis generation, made identifying patterns more important than strict control for multiple comparisons. Applying FDR could have led to missing potentially meaningful associations, particularly in the context of small effect sizes. Second, there was no clear basis for a hierarchical testing approach, as endpoints like myocardial infarction, stroke, and cardiovascular mortality were of equal clinical relevance, making prioritization for testing difficult. Third, the relatively low environmental exposure levels in Denmark raised concerns about overcorrection, which could obscure relevant findings with real-world implications. Lastly, we prioritized robust confounding control in fully adjusted models, emphasizing the careful selection of covariates to ensure precise estimates, rather than focusing on FDR corrections.

Supplemental Table 1. Cox proportional hazards models to estimate hazard ratios for the association between pollution exposure and secondary CVD outcomes, separated into VIVA and DANCAVAS cohorts, respectively.

|  | Events VIVA | VIVA Cohort, fully adjusted model 3  n = 17,759 | Events DANCAVAS | DANCAVAS Cohort, fully adjusted model 3  n = 8,457 |
| --- | --- | --- | --- | --- |
| All-cause mortality  Noise, L_den_ PM_2.5_ NO_2_ Warm-season O_3_  CO  SO_2_ | n = 3,591 | **1.100 (1.041 to 1.161)**  0.898 (0.860 to 0.938)  0.994 (0.950 to 1.041)  0.982 (0.944 to 1.022)  0.984 (0.951 to 1.019)  0.983 (0.961 to 1.005) | n = 230 | 0.828 (0.681; 1.008)  0.953 (0.825; 1.102)  1.003 (0.854; 1.178)  0.998 (0.867; 1.149)  1.002 (0.882; 1.138)  1.021 (0.962; 1.083) |
| Stroke  Noise, L_den_ PM_2.5_ NO_2_ Warm-season O_3_  CO  SO_2_ | n = 1,497 | **1.135 (1.043 to 1.234)**  1.018 (0.954 to 1.085)  1.014 (0.945 to 1.088)  0.998 (0.938 to 1.062)  1.025 (0.976 to 1.078)  1.015 (0.995 to 1.036) | n = 127 | 1.048 (0.812 to 1.354)  0.999 (0.824 to 1.211)  0.960 (0.767 to 1.200)  1.022 (0.843 to 1.239)  0.952 (0.790 to 1.147)  1.032 (0.967 to 1.101) |
| AMI  Noise, L_den_ PM_2.5_ NO_2_ Warm-season O_3_  CO  SO_2_ | n = 986 | 1.002 (0.900 to 1.115)  1.067 (0.985 to 1.155)  1.009 (0.925 to 1.101)  1.010 (0.936 to 1.091)  1.043 (0.982 to 1.107)  1.007 (0.979 to 1.035) | n = 73 | 0.936 (0.664 to 1.320)  0.965 (0.748 to 1.245)  0.940 (0.695 to 1.271)  1.097 (0.842 to 1.429)  1.017 (0.809 to 1.277)  0.868 (0.666 to 1.131) |
| CV mortality  Noise, L_den_ PM_2.5_ NO_2_ Warm-season O_3_  CO  SO_2_ | n = 758 | 1.038 (0.920 to 1.171)  0.935 (0.850 to 1.028)  0.994 (0.900 to 1.097)  0.984 (0.902 to 1.073)  0.965 (0.893 to 1.043)  0.983 (0.938 to 1.031) | n = 29 | 0.902 (0.520 to 1.563)  1.049 (0.708 to 1.554)  0.942 (0.593 to 1.496)  1.086 (0.719 to 1.640)  0.967 (0.671 to 1.395)  1.044 (0.920 to 1.184) |
| Revascularization  Noise, L_den_ PM_2.5_ NO_2_ Warm-season O_3_  CO  SO_2_ | n = 2,427 | 1.027 (0.960 to 1.099)  **1.114 (1.060 to 1.171)**  **1.061 (1.004 to 1.120)**  0.956 (0.911 to 1.003)  **1.063 (1.025 to 1.103)**  1.009 (0.993 to 1.026) | n = 249 | 0.997 (0.828; 1.199)  1.045 (0.909; 1.200)  0.977 (0.832; 1.146)  1.026 (0.892; 1.179)  1.014 (0.897; 1.146)  0.942 (0.845; 1.053) |
| Heart failure  Noise, L_den_ PM_2.5_ NO_2_ Warm-season O_3_  CO  SO_2_ | n = 1,098 | 0.973 (0.879 to 1.077)  1.053 (0.976 to 1.136)  1.033 (0.952 to 1.121)  0.976 (0.909 to 1.049)  1.034 (0.976 to 1.097)  1.006 (0.979 to 1.033) | n = 80 | 1.018 (0.739; 1.403)  1.090 (0.852; 1.393)  1.204 (0.934; 1.552)  0.866 (0.691; 1.087)  1.199 (1.023; 1.405)  0.860 (0.668; 1.106) |

*Hazard ratios for the secondary outcomes (all-cause mortality, stroke, AMI, CV mortality, revascularization, heart failure) per IQR increase in pollutant levels (traffic noise, PM_2.5_, NO_2_, warm-season* O_3_*, CO, SO_2_). The table shows HRs for the fully adjusted model three, separated into VIVA and DANCAVAS cohorts.*

Supplemental Figure 2. Dose-response curve for traffic noise and risk of CVD outcomes, separated into VIVA and DANCAVAS cohorts, respectively.

VIVA cohort


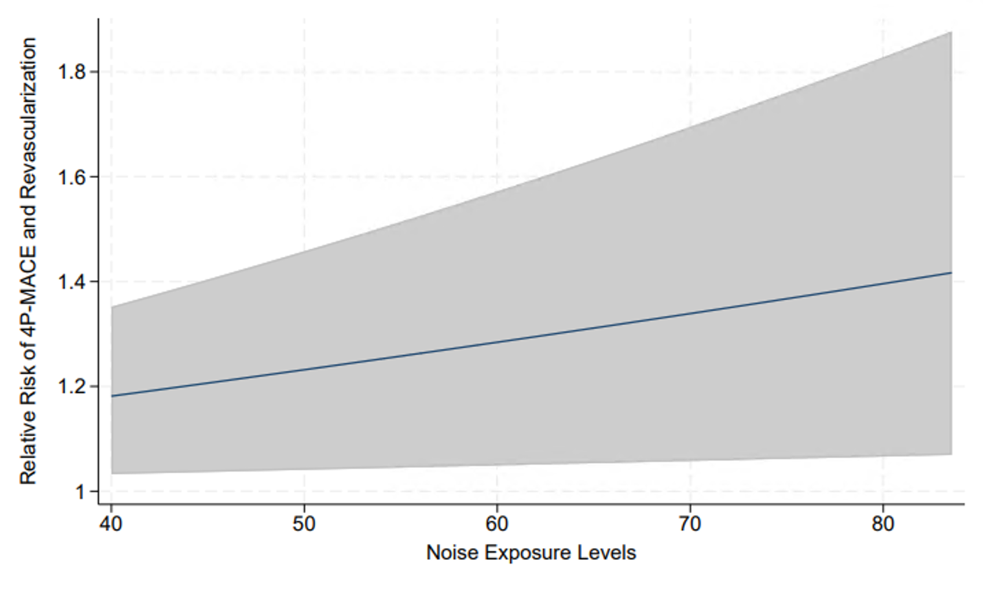


DANCAVAS cohort


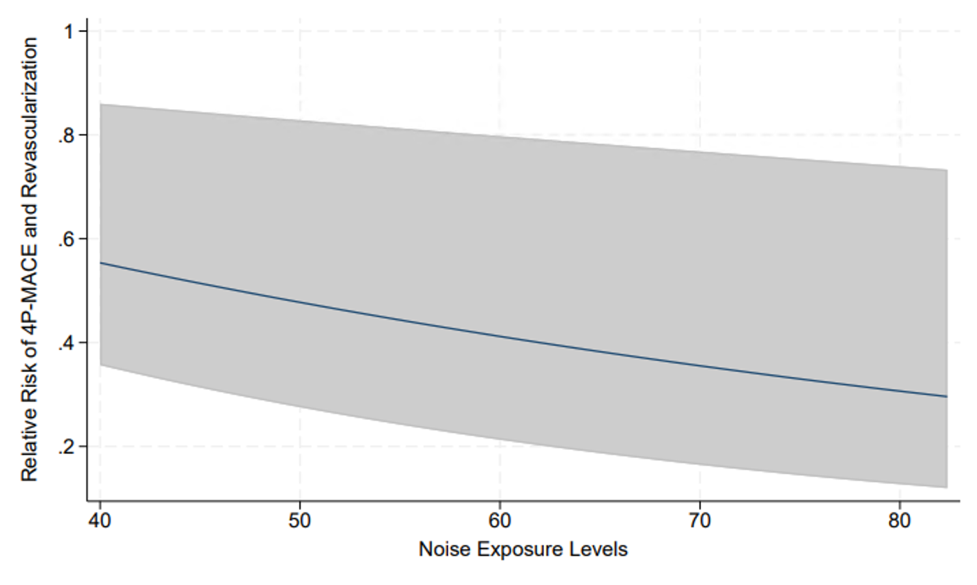


*Figure shows the dose-response curve illustrating the association between increasing levels of traffic noise in dB and the composite primary endpoint MACE & revascularization for both the VIVA and DANCAVAS cohort, respectively. The X-axis quantifies traffic noise levels in L_den_ in dB while the Y-axis measures the relative risk of experiencing an event. The solid line represents the calculated relative risk at increasing exposure levels. The surrounding shaded area shows the 95% confidence interval.*

Supplemental Figure 3. Dose-response curve for PM_2_**_._**_5_ exposure and risk of CVD outcomes, separated into VIVA and DANCAVAS cohorts, respectively.

VIVA cohort


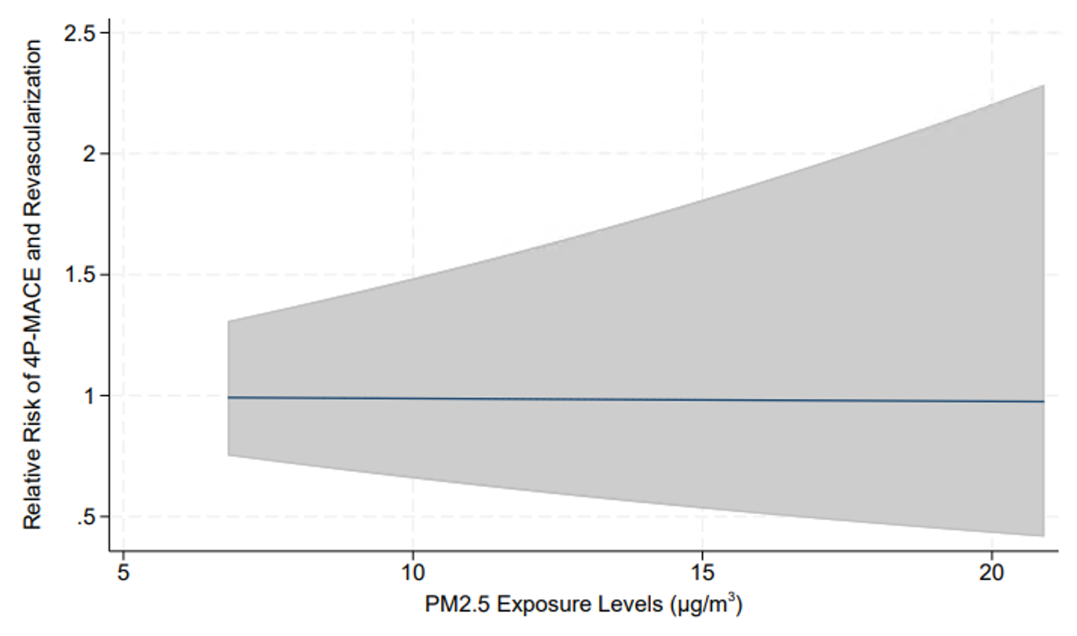


DANCAVAS cohort


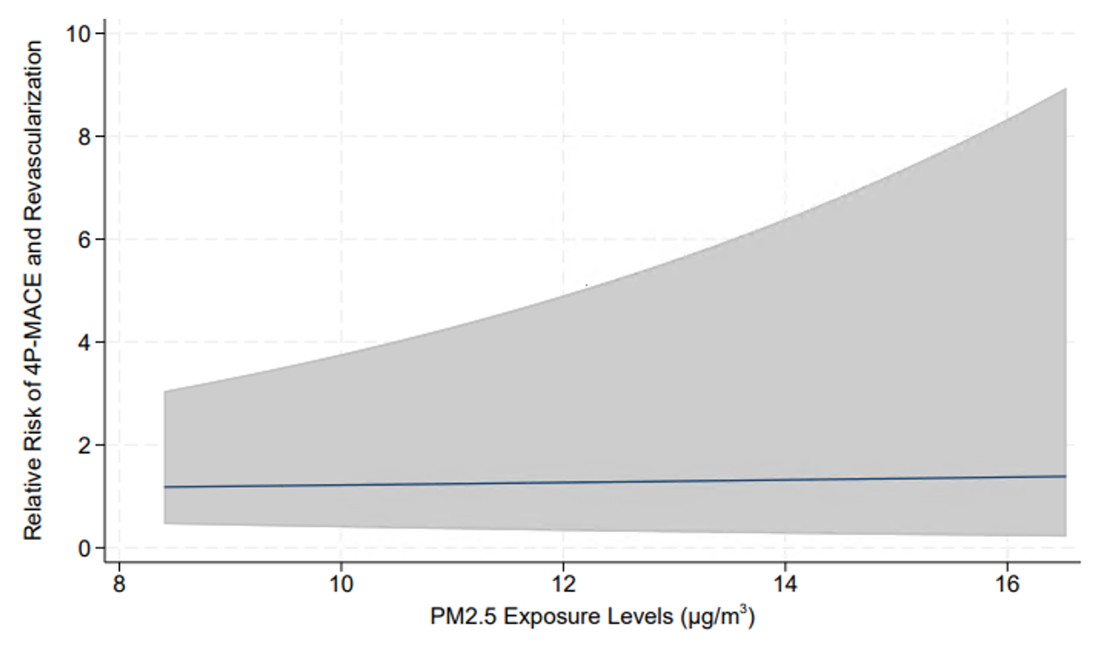


*Figure shows the dose-response curve illustrating the association between increasing levels of PM_2.5_ exposure in µg/m^3^ and the composite primary endpoint MACE & revascularization for both the VIVA and DANCAVAS cohort, respectively. The X-axis quantifies PM_2.5_ exposure levels in µg/m^3^ while the Y-axis measures the relative risk of experiencing an event. The solid line represents the calculated relative risk at increasing exposure levels. The surrounding shaded area shows the 95% confidence interval.*

# References

1. Giorgini P, Di Giosia P, Grassi D, Rubenfire M, Brook RD, Ferri C. Air Pollution Exposure and Blood Pressure: An Updated Review of the Literature. Curr Pharm Des. 2016;22(1):28-51.

2. McGuinn LA, Schneider A, McGarrah RW, Ward-Caviness C, Neas LM, Di Q, et al. Association of long-term PM(2.5) exposure with traditional and novel lipid measures related to cardiovascular disease risk. Environ Int. 2019;122:193-200.

3. Li Y, Xu L, Shan Z, Teng W, Han C. Association between air pollution and type 2 diabetes: an updated review of the literature. Ther Adv Endocrinol Metab. 2019;10:2042018819897046.
